# Influence of Clinker and Cinder Particle Gradation on the Properties of Blended Cement and Associated Mortars

**DOI:** 10.3390/ma18122864

**Published:** 2025-06-17

**Authors:** Runze Shang, Dexiang Huang, Wenju Cai, Longlong Niu, Bi Chen, Xinyu Zhang, Wei Li, Min Deng

**Affiliations:** 1 College of Materials Science and Engineering, Nanjing Tech University, Nanjing 211816, China; runze12318@163.com (R.S.);; 2 Hefei Cement Research & Design Institute Corp., Ltd., Hefei 230051, China; 3 Anhui Provincial Key Laboratory of Green and Low-Carbon Technology in Cement Manufacturing, Hefei 230051, China; 4 CNBM (Hefei) Powder Technology Equipment Corp., Ltd., Hefei 230051, China; 5 College of Engineering Management, Nanjing Tech University, Nanjing 211816, China

**Keywords:** grinding method, cement clinker, cinder, compressive strength, water requirement of standard consistency

## Abstract

The high-hydrolysis reactivity cement clinker powder in cement plays a major role in cement’s cementation, while low-hydrolysis reactivity mineral admixture powders, such as slag, m mainly serve as a filler. Through optimizing the particle matching of cement clinker powder and slag powder, the mechanical properties of cement can be enhanced. In this study, clinker and slag with differing levels of fineness were obtained by separate grinding, and the particle gradation of clinker powder and slag powder in the cement was optimized. Fine clinker particles were mixed with coarse slag particles to systematically explore their effects on the rheology of cement paste, the formation of hydration products, the evolution of the pore structure, and the material’s mechanical properties. Through experimental tests and microscopic analysis, the mechanism whereby particle gradation is regulated by separate grinding was revealed. The findings of the study are as follows: with the same amount of cinder, finer clinker requires a higher water content of standard consistency. The addition of coarse cinder effectively reduces the standard-consistency water requirement of the blended cement. Fine grinding of coal cinder fails to enhance cement strength effectively but markedly raises the standard-consistency water demand. Thus, the specific surface area of coal cinder should be maintained at approximately 210 m^2^/kg.

## 1. Introduction

In 2024, China’s total coal consumption was 5.96 billion tons, accounting for 53.2% of the country’s total energy consumption [1]. Chinese coal-fired power plants generate approximately 600–700 million tons of solid industrial residues annually, primarily comprising fly ash (about 80–90%) and coal-fired slag (about 10–20%). Fly ash comprises fine particulate matter collected from flue gases, whereas coal-fired slag refers to the coarser residues accumulated at the bottom of the furnace (also referred to as bottom ash or cinder) [2].

Fly ash has been widely adopted in industrial applications, particularly as a cementitious additive in concrete and construction materials, owing to its favorable pozzolanic properties and consistent particle morphology [3,4]. However, the potential application of cinder is fundamentally limited by its unstable structure and poor compaction characteristics, which seriously affect its functionality [5,6]. Therefore, only a limited fraction of cinder is utilized in subbase fill and brick manufacturing [7,8,9]. Over 85% of the cinder generated by coal-fired power plants is either stockpiled in open-air storage or disposed of in landfills, which has a significant impact on the surrounding air and soil [4,10,11]. Recent studies have confirmed the potential of cinder for use in construction applications. However, in China, its value-added utilization remains limited and is primarily restricted to brick production and road construction. Zhang et al. [12] prepared three different types of sintered bricks using lakebed mud, cinder, and sludge as the primary raw materials. Moreno Nabarro et al. [13], Fedorova and Shaforost [14], and other researchers have found that using cinder as a substitute for traditional road construction materials in road construction and maintenance applications can yield some economic benefits. In addition, cinder can be used to produce concrete [5,15]. Coarse aggregates can partially replace the stone, while fine aggregates can replace some of the sand and cement [16,17,18,19]. In recent years, much research has focused on using cinder to prepare concrete mixtures. However, bricks containing cinder are prone to cracking, and using cinder as a road base or fine aggregate fails to utilize its chemical properties, leading to resource waste. It is urgent to reasonably and fully develop and utilize cinder.

Cinder contains significant amounts of reactive components such as SiO_2_ and Al_2_O_3_, which enable its use as a supplementary cementitious material. This application can help reduce cement consumption and enhance its added value in construction materials. However, its low early-age reactivity limits the incorporation ratio, hindering large-scale application. Existing technologies improve its reactivity through superfine grinding, but this results in high energy consumption and costs. Therefore, finding ways to increase the cinder content while reducing costs and improving cement strength has become a key challenge.

Cement-based materials are among the most widely used structural materials in modern construction projects, and their mechanical properties have a significant impact on both the engineering quality and durability. As core components, cement clinker and cinder not only influence the process of the hydration reaction but also directly determine the microstructure of the paste and its mechanical properties.

Composite cement is typically produced by blending Portland cement with supplementary cementitious materials (SCMs), including ground granulated blast-furnace slag, fly ash, or steel slag. Currently, the content of high-activity waste residues (such as slag and fly ash) in composite cement can reach 20–30%, while the content of low-activity waste residues (such as cinder and steel slag) is usually below 10% [20,21,22,23]. This preparation method is relatively simple, but the utilization of SCMs is low. When the content of these materials is higher, the early strength and other properties of the blended cement are poor, which limits the amount of SCMs that can be incorporated into the blended cement.

In addition, analyzing the particle size distribution of cement using the Rosin–Rammler–Bennet (RRB) distribution and the Fuller ideal packing curve [24,25] reveals that an insufficient amount of fine cement particles makes it difficult for the grinding system to achieve the ideal packing curve in a single process. Even when energy consumption is not considered, achieving the Fuller ideal packing curve through particle selection and blending would still lead to increased water demand, reduced early strength, poorer workability, and shrinkage cracking issues. This is evidently impractical and unsuitable for guiding production. Therefore, researchers recommend using a graded grinding process, where SCMs replace some of the fine cement particles. This approach not only optimizes the particle packing density but also mitigates the adverse effects of excessively fine cement particles [26,27].

In recent years, a large number of scholars have focused on the ultrafine grinding of low-grade SCMs, solid waste, or industrial by-products, and their subsequent blending with cement clinker. Kupwade-Patil et al. replaced Portland cement with finer volcanic ash components obtained through grinding and found that the finer volcanic ash components produced a more compact pore structure in the paste, thereby achieving higher compressive strength [28]. Carvalho et al. conducted experiments and found that replacing 25% of Portland cement with fine recycled powder in cement mortar resulted in almost the same compressive strength as the control group mortar [29]. Other researchers have found that, when the replacement rate of fine steel slag does not exceed 20%, its effect on the strength and drying shrinkage of ordinary concrete can be considered negligible [30]. Bui et al. found that, compared to the combination with fine cement, the composite of rice husk ash with coarse cement resulted in a higher increase in the relative strength of the paste [31]. Pormmoon et al. [32] found that micronized high-calcium and low-calcium coal bottom ash (D50 ≈ 4 μm) significantly improved the 28-day compressive strength of high-performance concrete while reducing chloride permeability and water adsorption. This was attributed to the filler effect of fine particles and pore refinement via pozzolanic reactions. Liew et al. [33] systematically studied the synergistic gradation effects of low-calcium coal bottom ash (LCBA) and ground granulated blast-furnace slag (GGBS). When 15% cement was replaced by LCBA combined with GGBS, the ternary blend achieved 58.1 MPa compressive strength (52.5 N grade) with 45% lower CO_2_ emissions. These studies confirm that particle gradation optimization is critical for enhancing cement’s mechanical properties and durability. They also propose a sustainable strategy: balancing performance and environmental benefits by tailoring fineness and calcium content [32,33]. Thus, integrating coal-based waste with clinker and slag, guided by particle design, could optimize cement performance while reducing environmental impact.

Despite extensive research on the use of cinder in cementitious materials, several critical gaps remain. First, most studies focus on ultrafine grinding of cinder to enhance reactivity, which incurs high energy costs without proportional strength gains. Second, the synergistic effects of particle gradation between clinker and cinder on hydration kinetics and pore structure evolution are poorly understood. Third, existing work rarely addresses the trade-off between water demand and mechanical properties when optimizing particle size distributions. This study fills these gaps by (1) proposing a cost-effective strategy combining fine clinker with coarse cinder to balance reactivity and water requirements, (2) systematically linking particle gradation to microstructure development through hydration heat and pore analysis, and (3) establishing an optimal fineness ratio (clinker: 498 m^2^/kg; cinder: 210 m^2^/kg) that outperforms conventional blends in terms of both strength and workability. These contributions advance the sustainable utilization of cinder while reducing grinding energy consumption.

## 2. Materials and Methods

### 2.1. Raw Materials

**Figure 1:**
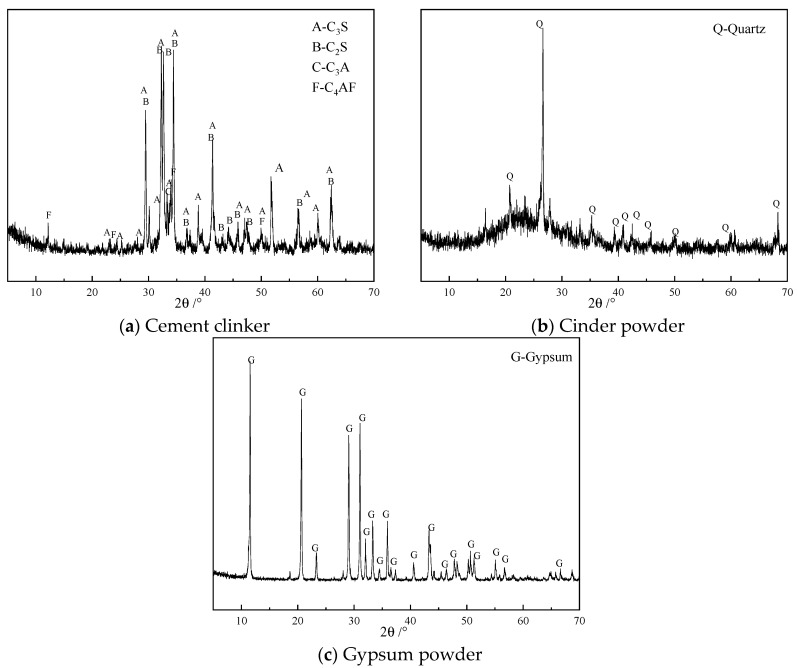
X-ray diffraction (XRD) pattern of cement clinker, cinder powder, and gypsum powder.

All raw materials, including cement clinker, cinder, and gypsum, were sourced from Hefei Southern Cement Co., Ltd., (Hefei, China). The mineral compositions of cement clinker and cinder were characterized by X-ray diffraction (XRD) following Chinese standard JY/T 0587-2020 [34]. The X-ray powder diffraction data were collected at room temperature using a Rigaku Smart Lab 3 kW diffractometer, Rigaku, Tokyo, Japan, and the scanning speed was 5°/min. Figure 1 shows the results. The mineral composition of cement clinker primarily consists of C_2_S, C_3_S, C_3_A and C_4_AF, while the cinder is mainly composed of vitreous material, containing small amounts of quartz and mullite.

Cement clinker C was produced by Hefei South Cement Co., Ltd., (Hefei, China). The cinder and gypsum used were also from Hefei South Cement Co., Ltd. The chemical composition was determined following Chinese National Standard GB/T 176-2017 [35] for cement analysis. Table 1 shows the results. The main chemical components of the cinder are SiO_2_, Al_2_O_3_, and Fe_2_O_3_. The primary chemical components of the gypsum are SO_3_ and CaO.

### 2.2. Methods

#### 2.2.1. Specific Surface of Cement—Blaine Method

The specific surface area was measured using an SBT-127 digital Blaine permeability (Wuxi, China) apparatus in accordance with GB/T 8074-2008 [36]: “Testing method for specific surface of cement—Blaine method”.

#### 2.2.2. Water Requirement of Standard Consistency

The test determines the water quantity needed for cement paste to reach 6 ± 1 mm plunger penetration in a Vicat apparatus, following GB/T 1346-2024 [37]. It mixes cement with varying water percentages until the standard penetration depth is achieved.

#### 2.2.3. Mechanical Strength

The cement strength was determined according to GB/T 17671-2021 [38]. The experiment utilized standard sand with a water-to-cement ratio of 0.50. The specimens were cured under standardized conditions (20 °C, 95% relative humidity), and the compressive strength of the specimens was measured at curing ages of 3 days and 28 days.

#### 2.2.4. Preparation of Cement Paste Samples

In the microstructure testing of cement paste, a cement paste with a water-to-binder ratio of 0.3 is prepared. The paste is sealed and cured in an environment at 20 °C. After curing for the specified age, the paste is removed and placed in alcohol to terminate the hydration before testing.

#### 2.2.5. Heat of Hydration

The cement powder and water were first placed in a 20 °C environment to ensure that the material temperature matched the measurement ambient temperature. The heat of hydration was analyzed using the TAM AIR Thermstate90 calorimeter, manufactured by TA Instruments (New Castle, DW, USA), with a water-to-cement ratio of W/C = 0.5. The sample mass was approximately 5.00 g, and the temperature was maintained at a constant 20 °C.

#### 2.2.6. Cement Paste Pore Structure

Mercury intrusion porosimetry was performed using the Micromeritics AutoPore IV 9610 (Norcross, GA, USA) instrument from the United States, which is one of the primary tools for testing the pore structure of cement paste. Prior to the pore structure analysis, the specimen was dehydrated in a vacuum oven at 105 °C for 4–5 h to eliminate capillary water and residual alcohol. Mercury intrusion porosimetry was then employed, where pressurized mercury was progressively forced into the accessible pores of the cement matrix while simultaneously recording both the intrusion pressure and cumulative mercury volume. This method has a test range of 7 nm to 200 μm.

## 3. Results and Discussion

### 3.1. Processing of Raw Materials

When grinding cement clinker with roller-ball mill, the granular clinker is crushed to a particle size less than 8 mm, and then it is ground and prepared by the laboratory roller press–mill–ball mill–powder concentrator system. Table 2 shows the specific surface area, sieve residue, and particle size of clinker and gypsum powder produced. The sample numbers in the table are represented by the raw material number followed by the D_90_ value (in μm).

**Figure 2:**
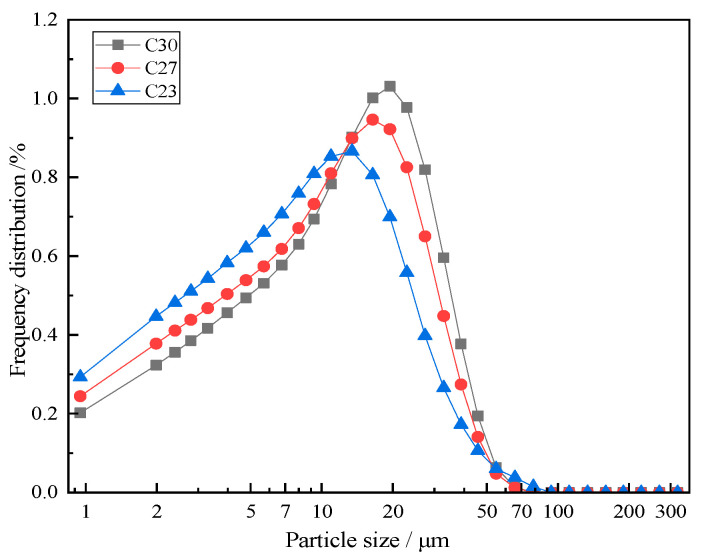
Frequency distribution of cement clinker powder.

Figure 2 shows the particle size distribution of cement clinker. The frequency distribution of clinker particles ground in a roller press shows only one peak particle size and follows a skewed normal distribution.

**Figure 3:**
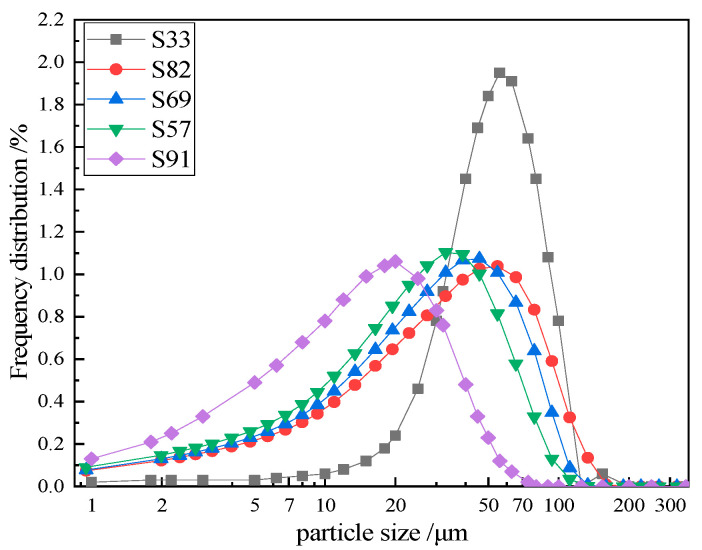
Frequency distribution of cinder powder particles.

By altering the powdering time and the rotational speed of the powder concentrator, five kinds of cinder powders with specific surface areas of approximately 106 m^2^/kg, 210 m^2^/kg, 260 m^2^/kg, 300 m^2^/kg, and 445 m^2^/kg were obtained. Table 3 shows the specific surface area, sieve residue, and particle size distribution of the cinder. Figure 3 shows the laser particle size distribution of the cinder powder. The results indicate that the particle size distribution of the cinder powder exhibits only one peak particle size and follows a skewed normal distribution. The maximum observed particle diameter was below 200 μm, with the >45 μm fraction from negative pressure sieving showing an excellent correlation with laser diffraction measurements.

The experiment used finer clinker powder, coarser cinder, and gypsum powder to create cement. The cinder powder was mixed at ratios of 15%, 20%, and 25%. The gypsum content in all cements was 5% across a total of 11 kinds of samples. The cement samples were labeled with the first letter of the clinker (C) + the D_90_ of the clinker powder + the first letter of the cinder (S) + the D_90_ of the cinder + the cinder content (in %). For example, C30S82-15 represents a mixture of clinker powder with a D_90_ of 30 μm and cinder powder with a D_90_ of 82 μm, with the cinder content being 15%.

### 3.2. Specific Surface Area and Fineness of Cement

Table 4 shows the specific surface area of cement prepared with clinker and cinder powders. When the cinder powder content was 15%, 20%, and 25%, the specific surface areas of the cements were 342–460, 337–448, and 332–438 m^2^/kg, respectively. The content of particles larger than 45 μm was 6.01–8.47%, 7.27–10.19%, and 8.04–12.21%, respectively.

### 3.3. Uniformity Coefficient of Cement

Cementitious materials exhibit complex particle size distributions. Experimental data indicate that most cement systems conform to the Rosin–Rammler–Bennet (RRB) distribution. The RRB equation’s uniformity coefficient (n) effectively characterizes particle size distribution across varying fineness levels. The RRB equation is expressed as:

(1) $$R(D_{P})=100exp\;[-{\left(\frac{D_{P}}{D_{e}}\right)}^{n}]$$

where *R*(*D_p_*) represents the cumulative oversize percentage (%), *D_p_* denotes the particle diameter (μm), and *D_e_* is the characteristic particle size constant. The exponent n describes the distribution breadth, with smaller n values indicating broader distributions

Table 5 shows the uniformity coefficient (n) values for the blended cements. The results show that the n values for cinder-blended cements range from 0.932 to 1.132. The uniformity coefficient is smaller for blends of fine clinker (C23) and coarse cinder (S82), indicating a wider particle size distribution.

### 3.4. Water Requirement of Standard Consistency for Cement

**Figure 4:**
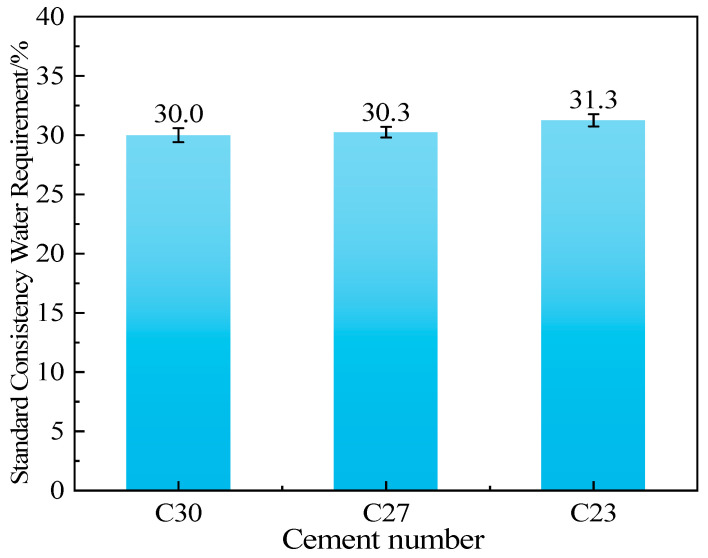
Water requirement of standard consistency for clinker-blended cement pastes.

**Figure 5:**
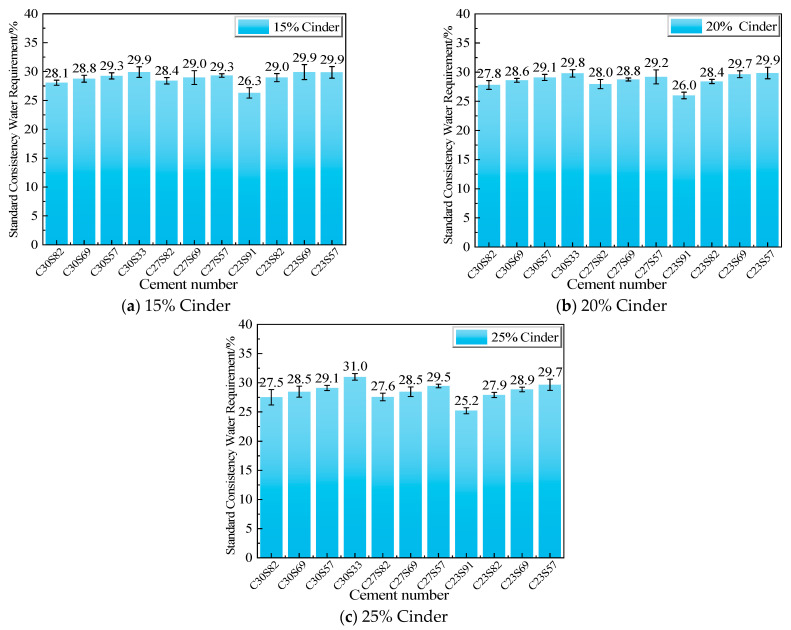
Water requirement of standard consistency for cement pastes blended with 15–25% cinder content.

Figure 4 shows the water requirement of standard consistency for clinker-based blended cement pastes. Figure 5 shows the standard-consistency water requirement of cement pastes with added cinder. As the fineness of the cinder increases, the water requirement of the cement pastes increases. At the same cinder content, different blending methods result in significant differences in water requirements. The water requirement is relatively small for the blend of coarse cinder and clinker. The inverse correlation between cinder fineness and water demand matches findings from Pornmoon et al. [32], who noted a 15% increase in water demand for micronized cinder (D_50_ ≈ 4 μm). However, our results demonstrate that coarser cinder (D_50_ ≈ 31 μm) reduces the water requirement by 12–18%, surpassing the 8–10% improvement reported by Liew et al. [33] for GGBS blends. For the same cinder content, the cement with a C30S82 ratio generally has a smaller water requirement, while the cement with a C23S57 ratio has a larger water requirement.

Table 6 shows the minimum standard consistency water requirements for cement pastes blended with 15–25% cinder, along with their corresponding specific surface area and uniformity coefficient (n) values. The results indicate that, for the same amount of cinder, the blending of coarse cinder with coarse clinker results in a smaller water requirement.

When the amount of cinder is the same, the finer the clinker is, the greater the standard consistency water requirement is. Adding coarse cinder can effectively reduce the standard consistency water requirement of the mixed cement. Moreover, as the amount of cinder increases, the fineness decreases, and the effect of reducing the standard consistency water requirement of the mixed cement becomes more significant.

The fineness of the cement clinker has a significant influence on the standard water requirement of cement. After cement and water are mixed, the water must first fill the gaps between the particles and wet the surfaces of the particles to form a water film on the surface, enabling relative sliding between particles and thus achieving better fluidity. The finer the cement clinker particles are, the larger the specific surface area is, and the contact area with water increases significantly. This leads to an increase in the amount of water required to cover the particle surfaces and fill the gaps, resulting in an increase in the standard water requirement. Generally, the larger the particles are, the thicker the water film must be to achieve better fluidity. The powder particles with a larger n value require a thicker water film [25]. As shown in Table 5 and Figure 5, for samples with similar specific surface areas, as the n-value increases, the packing void ratio increases, requiring more free water to fill these voids, thus increasing the standard consistency water requirement. Therefore, when the amount of cinder added is the same, the higher the uniformity coefficient is, and the greater the standard consistency water requirement will be. 

### 3.5. Strength of Compound Cement Mortar

The strength results of clinker with a D_90_ of 30, 27, and 23 μm (with 5% desulfurized gypsum) are shown in Table 7. The 28-day flexural strengths are 8.6, 8.5, and 8.2 MPa, and the 28-day compressive strengths are 59.0, 60.3, and 59.2 MPa, respectively. The cement fineness has little effect on the 28-day compressive strength.

**Figure 6:**
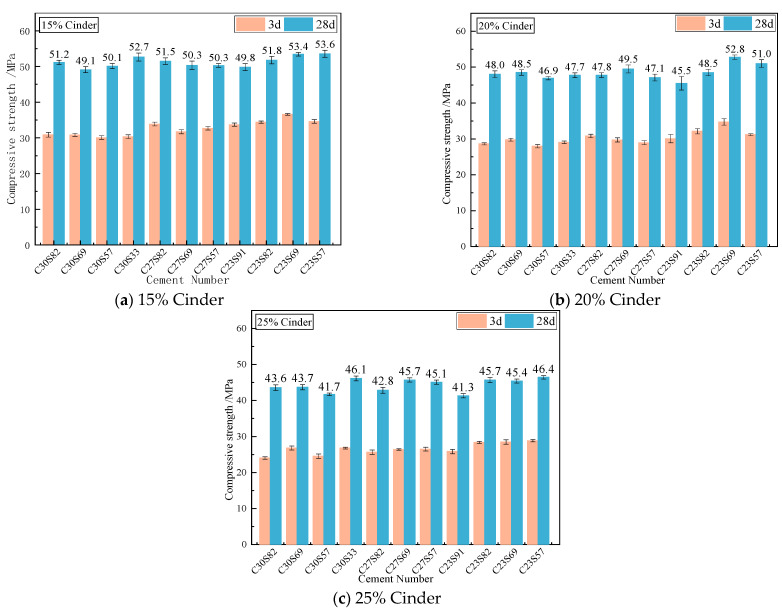
Compressive strength of cement mortar with 15–25% cinder.

Figure 6 shows the strength of cement with 15–25% cinder. The differences in the 28-day compressive strengths of cement mixed with 15%, 20%, and 25% cinder are 4.5, 5.8, and 4.7 MPa, respectively. In the cement mixtures with different fineness levels in their clinker and slag, the strength of C23S91 always remains relatively low under different production volumes. This indicates that the slag particles should not be too coarse. However, in the C23 specimens, the influence of different slag fineness levels on the 28-day compressive strength is relatively small. The limited strength gain from fine cinder corroborates the findings of Carvalho et al. [25], who found that recycled powder fineness beyond 300 m^2^/kg had diminishing returns. This suggests that grinding coal slag finely cannot effectively enhance the strength of cement but instead will significantly increase the water requirement of the standard consistency. Therefore, considering both strength and water requirements, the specific surface area of coal slag should be controlled at around 210 m^2^/kg.

Table 8 shows the cement samples with the best comprehensive performance. By comparing the fluidity and mechanical properties of cement, the optimal mixture ratio of cement with the best comprehensive performance is selected. The selection criterion for the best comprehensive performance is mainly based on the 28-day compressive strength, with the water content of standard consistency as a secondary indicator. Specifically, the 28-day compressive strength of each group of specimens is ranked from high to low. If the compressive strengths are similar, the standard consistency water content is further compared to select the one with lower water content to optimize workability and economy. Finally, the material with the best comprehensive performance should have both high mechanical properties and low water consumption. The combination of cement with 15–25% cinder content that achieves the best overall performance is all C23S82.

### 3.6. Hydration and Microstructure of Cement Paste

#### 3.6.1. Hydration Products of Cement

**Figure 7:**
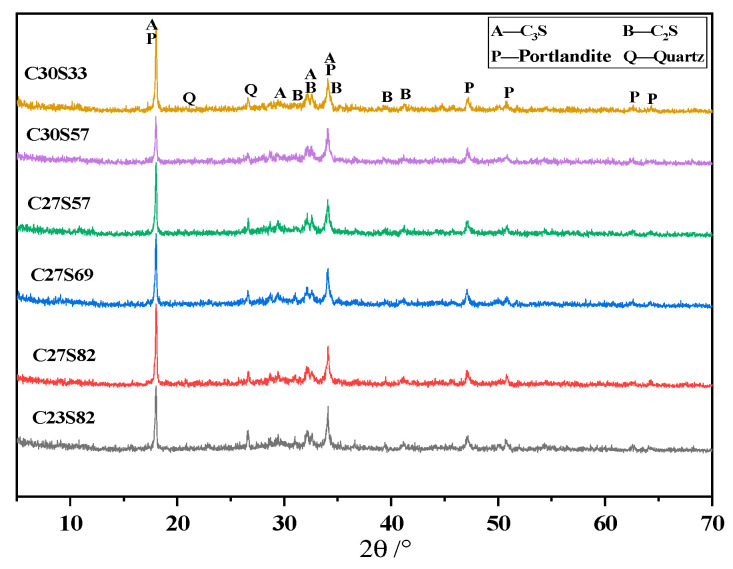
XRD pattern of the 28-day curing of cement pastes with cinder compound.

Figure 7 shows the XRD spectra of cement pastes with 20% cinder after curing for 28 days. After 28 days of hydration, the cement paste primarily contains C2S, C3S, and calcium hydroxide. The diffraction peaks of C_2_S and C_3_S in the cement paste are relatively strong, indicating that some unhydrated clinker still remains after 28 days of hydration.

#### 3.6.2. Compound Cement Hydration Heat

**Figure 8:**
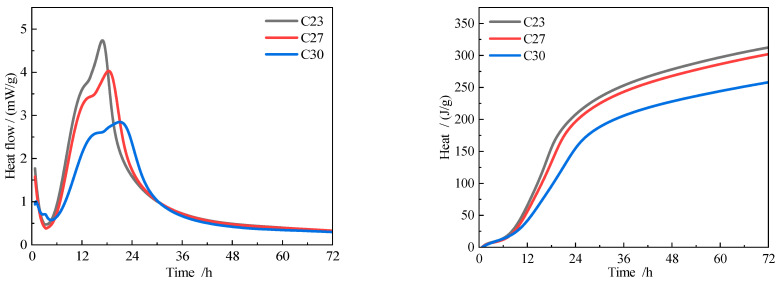
Hydration exothermic curves of cement.

Figure 8 shows the hydration heat of pure cement. The finer the cement clinker, the earlier the second exothermic peak appears and the higher the heat release rate. The cement samples C23, C27, and C30 achieved maximum heat release rates of 4.7 mW/g, 4.0 mW/g, and 2.8 mW/g, respectively. The cumulative heat release after 72 h reached 312 J/g, 302 J/g, and 257 J/g, respectively. The earlier second exothermic peak in C23 blends reflects the accelerated nucleation of C-S-H due to higher clinker surface area. However, coarse cinder (S82) mitigates excessive heat release by diluting reactive sites, reducing cracking risk. This synergy—fine clinker providing nucleation centers and coarse cinder acting as a thermal buffer—explains the optimal performance of C23S82.

**Figure 9:**
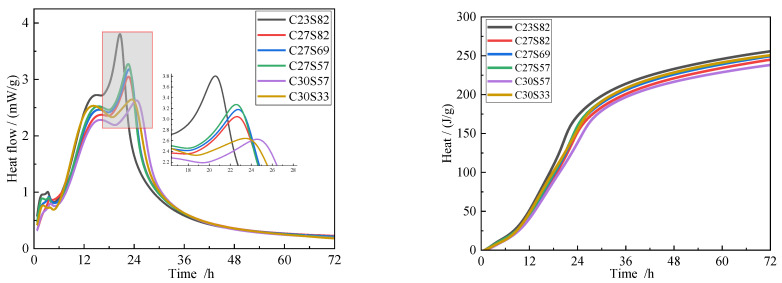
Hydration exothermic curves of cement blended with 20% cinder.

Figure 9 shows the hydration heat of cement with 20% cinder. The experimental results show that the second exothermic peak of the cement primarily occurs around 24 h, and the incorporation of slag significantly reduces the heat release rate of this peak. Comparing samples with different slag contents, the second exothermic peak of C30S33 appears slightly earlier than that of C30S57, though the cumulative heat release at 72 h for both samples shows no significant difference. This suggests that fine slag promotes the hydration reaction of blended cement in the early stages (within 24 h) but has a minimal impact on the hydration process in the later stages (after 72 h). This phenomenon may be related to the micro-filler effect of the slag and the time-dependent activation of its latent reactivity. The pore refinement in C23S82 (Table 9) arises from two mechanisms: (1) fine clinker fills inter-cinder voids (physical effect), and (2) its rapid hydration generates secondary C-S-H that seals capillary pores (chemical effect). Coarse cinder’s lower surface area limits water adsorption, preserving workability without compromising densification, as observed in similar studies [32,33].

#### 3.6.3. Compound Cement Hole Structure

**Figure 10:**
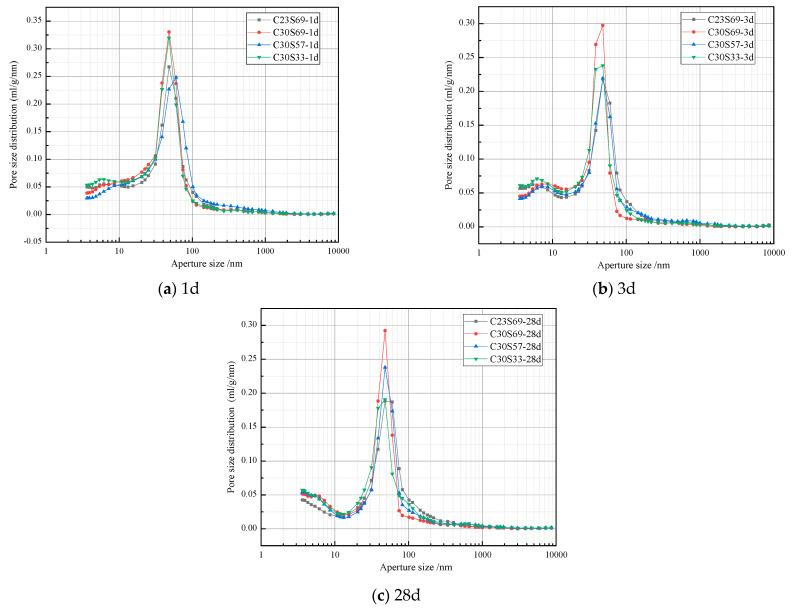
Pore size distribution of cinder cement pastes (1d, 3d, 28d).

The mechanical strength, shrinkage behavior, and pore structure of cement pastes are closely related. Previous research classifies the pore size (d) of cement paste into four distinct categories based on their detrimental effects: pores below 20 nm, which are deemed harmless; pores ranging from 20 to 50 nm, classified as minimally harmful; pores between 50 and 200 nm, considered harmful; and pores exceeding 200 nm, which are highly harmful. Table 9 shows the porosity of hardened cement paste mixed with 20% cinder. As cement fineness and cinder fineness increase, the porosity at 28 days continuously decreases. The pores of 0 to 50 nm gradually increase, while the pores of >50 nm gradually decrease. Specifically, the paste of C30S33 exhibits the lowest total porosity. Compared to C30S69, its porosity decreases from 22.82% to 21.83%, with a reduction of only 1%. This result indicates that, although fine slag can optimize the pore structure, its effect on reducing total porosity is limited, likely only contributing a mild physical filling effect. Figure 10 shows the pore size distribution of the cement paste with different clinker fineness and 20% cinder incorporation. The results show that the peak of the pore distribution in the cement paste appears in the 10–200 nm range. The most significant pore size in the cement paste is between 40–50 μm, exhibiting a distinct unimodal distribution characteristic.

#### 3.6.4. The Specific Microscopic Structure of Cement

**Figure 11:**
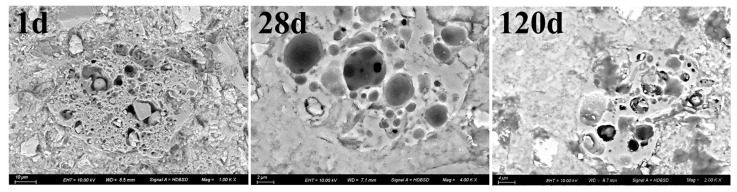
BSEM image of C27S69 cement pastes after being hydrated for 1d, 28d and 120d.

Figure 11 shows BSEM images of C27S69 cement pastes at 1, 28, and 120 days of hydration. At 1 day, the hydration products around cinder particles appear loose and porous, indicating limited early reactivity. By 28 days, the microstructure becomes denser, with reduced porosity due to C-S-H gel formation. At 120 days, further densification occurs, and unhydrated clinker particles diminish significantly, confirming the long-term role of coarse cinder as a stable microaggregate. These observations align with the measured reduction in harmful pores (>50 nm) from 30.8% (1d) to 22.8% (28d) in Table 9.

**Figure 12:**
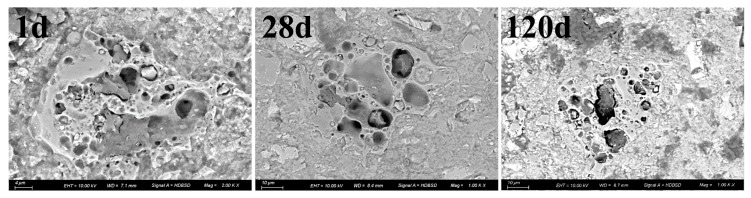
BSEM image of C30S57 cement pastes.

Figure 12 (C30S57 paste) reveals a compact ITZ between cinder particles and the cement matrix, with no visible cracks or voids. This contrasts with typical ITZs in fine-cinder blends (e.g., C30S33), where high water demand often leads to microcracking. The dense ITZ explains the superior mechanical performance of C30S57 (48.5 MPa at 20% cinder) compared to finer-cinder mixes, as it ensures effective stress transfer.

**Figure 13:**
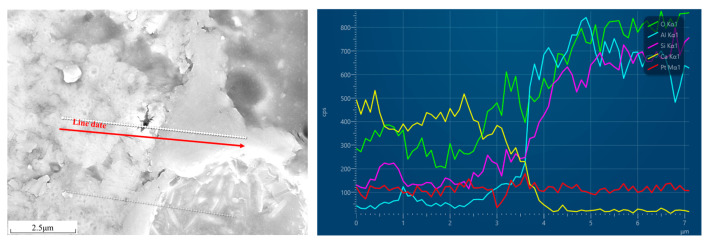
BSEM/EDS line scanning of C27S69 cement pastes after being hydrated for 120d.

The EDS line scanning in Figure 13 (C27S69 at 120 days) shows uniform Ca and Si distribution around cinder particles, confirming their integration into the hydration products. Notably, Al and Fe signals from cinder remain localized, indicating minimal chemical dissolution. This supports the conclusion that coarse cinder primarily acts as a physical filler, while fine clinker drives hydration.

## 4. Conclusions

This study systematically investigated the influence of clinker–cinder particle gradation on the properties of blended cement through controlled grinding and mixing strategies. The key findings and their implications are summarized as follows:

1. Blending fine clinker (specific surface area: 498 m^2^/kg) with coarse cinder (210 m^2^/kg) achieved the best balance between mechanical strength and workability. While finer cinder increased water demand by 15–20%, it provided negligible strength improvements (<5%). This highlights the importance of prioritizing coarser cinder to reduce water requirements without sacrificing performance.
2. Coarse cinder (D_90_ ≈ 91 μm) reduced the standard consistency water requirement by 12–18% compared to fine cinder (D_90_ ≈ 33 μm), owing to its lower surface area and improved particle packing efficiency. This effect was amplified at higher cinder contents (25%), suggesting its suitability for high-volume SCM applications.
3. Cement blended with fine clinker and coarse cinder hydrates faster within the first day and reaches a higher degree of hydration, which is more beneficial for pore filling. Due to the initial packing voids of the particles, the porosity of the C30S33 blended cement paste after 28 days of curing was relatively low. Although the hydration heat results show that fine cinder benefits the hydration of cement, the effect is limited, and the contribution to strength is not significant.

This research provides an actionable framework for optimizing blended cements: (1) maximize clinker fineness to drive hydration, (2) limit cinder fineness to ~210 m^2^/kg to control water demand, and (3) target a uniformity coefficient (n) of 0.93–0.98 for optimal packing.

## Figures and Tables

**Table 1:** Chemical composition of cement clinker and cinder.

Sample	CaO	SiO_2_	Fe_2_O_3_	Al_2_O_3_	MgO	K_2_O	Na_2_O	SO_3_	Loss	Total
Clinker C	64.97	21.18	3.75	6.23	1.51	0.28	0.48	0.71	0.56	99.67
Cinder S	6.34	58.85	6.16	23.02	0.76	1.29	0.91	0.30	0.70	98.33
Gypsum G	32.52	1.48	0.13	0.50	0.32	0.12	0.15	43.46	21.10	99.78

**Table 2:** Specific surface area, sieve residue, and particle size of clinker and gypsum powder ground.

Numbering		C30	C27	C23	G
Specific Surface Area (m^2^/kg)		368	422	498	174
Negative pressure sieve residue (%)	80 μm	0.0	0.0	0.0	4.1
	45 μm	0.0	0.0	0.0	43.4
Particle size (μm)	D_10_	1.66	1.46	1.3	3.76
	D_50_	10.96	9.15	7.13	31.06
	D_90_	29.49	27.02	23.21	61.53

**Table 3:** Specific surface area, sieve residue, and particle size of the cinder.

Numbering		S91	S82	S69	S57	S33
Specific Surface Area (m^2^/kg)		106	213	252	289	445
Negative pressure sieve residue (%)	80 μm	7.6	10.0	3.5	1.6	0.0
	45 μm	62.3	42.0	31.1	17.8	0.0
Particle size (μm)	D_10_	23.86	4.36	4.07	3.52	2.39
	D_50_	52.76	31.59	27.47	23.15	12.81
	D_90_	90.96	81.73	68.92	57.44	33.13

**Table 4:** Specific surface area of cement prepared with clinker and cinder powders.

No	The D_90_ (μm) of Clinker (C) Powder	The D_90_/(μm) of Cinder (S) Powder	Cement Preparation Number	The Specific Surface Area of the Prepared Cement (m^2^/kg)		
				15%S	20%S	25%S
1	30	82	C30S82	342	337	332
2		69	C30S69	348	343	342
3		57	C30S57	360	351	351
4		33	C30S33	379	387	391
5	27	82	C27S82	385	373	367
6		69	C27S69	387	384	371
7		57	C27S57	402	393	386
8	23	91	C23S91	433	414	386
9		82	C23S82	441	434	420
10		69	C23S69	447	445	424
11		57	C23S57	460	448	438

**Table 5:** The uniformity coefficient (n) values for blended cements.

No	The D_90_ (μm) of Clinker (C) Powder	The D_90_ (μm) of Cinder (S) Powder	Cement Preparation Number	The Uniformity Coefficient (n) of the Prepared Cement		
				15%S	20%S	25%S
1	30	82	C30S82	1.0417	1.0433	1.0358
2		69	C30S69	1.0685	1.0579	1.0489
3		57	C30S57	1.1078	1.1073	1.1202
4		33	C30S33	0.9978	0.9965	0.9962
5	27	82	C27S82	0.9858	0.9907	0.9866
6		69	C27S69	1.0203	1.0069	0.9989
7		57	C27S57	1.0519	1.0503	1.0472
8	23	91	C23S91	0.9881	0.9832	0.9812
9		82	C23S82	0.9340	0.9197	0.9191
10		69	C23S69	0.9398	0.9347	0.9526
11		57	C23S57	0.9851	0.9834	0.9823

**Table 6:** The minimum standard consistency water requirements of cinder-blended cement.

Cinder Dosage	Minimum Water Requirement of Standard Consistency (%)	Sample	Specific Surface Area (m^2^/kg)	Uniformity Coefficient
15%	26.2	C23S91	433	0.988
20%	26.0	C23S91	414	0.983
25%	25.2	C23S91	386	0.981

**Table 7:** Flexural and compressive strength of clinker.

No	Clinker Number	Compressive Strength (MPa)		Flexural Strength (MPa)	
		3d	28d	3d	28d
1	C30	6.3	8.6	36.3	59.0
2	C27	6.7	8.5	37.9	60.3
3	C23	6.6	8.2	40.5	59.2

**Table 8:** Cement sample with the best comprehensive performance.

Cinder Dosage	The Sample with the Best Comprehensive Performance	28-Day Compressive Strength (MPa)	Water Requirement of Standard Consistency (%)
15%	C23S82	51.8	29.0
20%	C23S82	48.5	28.4
25%	C23S82	45.7	27.9

**Table 9:** Porosity and pore size distribution of cinder cement pastes.

Sample	Age (d)	Porosity (%)	Pore Size Distribution (%)				
			<10 nm	10–20 nm	20–50 nm	50–200 nm	>200 nm
C23S69	1	27.41	13.42	9.20	28.61	28.52	20.26
	3	25.90	15.48	8.30	25.78	26.30	24.14
	28	21.96	10.00	5.01	26.11	34.60	24.27
C30S69	1	30.80	11.22	9.98	33.40	24.53	20.88
	3	26.72	14.96	10.46	39.45	12.08	23.06
	28	22.82	14.00	4.99	34.14	22.07	24.81
C30S57	1	29.96	9.29	9.27	24.20	32.43	24.81
	3	25.82	14.71	9.54	27.99	23.83	23.93
	28	22.66	13.27	4.09	26.69	27.84	28.11
C30S33	1	29.15	14.62	10.37	34.99	24.12	15.91
	3	26.47	16.78	9.56	34.86	15.03	23.76
	28	21.83	14.27	5.73	34.68	20.41	24.91

## Data Availability

The data presented in this study are available on request from the corresponding author. The data are not publicly available due to privacy.
